# Gradual changes in model shape affect egg-directed behaviours by parasitic shiny cowbirds *Molothrus bonariensis* in captivity

**DOI:** 10.1098/rsos.221477

**Published:** 2023-05-10

**Authors:** Ignacio Crudele, Mark E. Hauber, Juan C. Reboreda, Vanina D. Fiorini

**Affiliations:** ^1^ Departamento de Ecología, Genética y Evolución and IEGEBA-UBA-CONICET, Facultad de Ciencias Exactas y Naturales, Universidad de Buenos Aires, Pabellón II Ciudad Universitaria, Buenos Aires C1428EGA, Argentina; ^2^ Department of Evolution, Ecology, and Behavior, School of Integrative Biology, University of Illinois, Urbana-Champaign, IL 61801, USA

**Keywords:** egg pecking, egg shape recognition, obligate brood parasitism, three-dimensional printing

## Abstract

Eggs are critically important for avian reproduction as all birds are oviparous. Accordingly, the recognition and care of own eggs represent the cornerstones of avian breeding, whereas the elimination of foreign objects, including brood-parasitic eggs and non-egg items from the nest are known to also increase fitness by refocusing incubation effort on the parents' own eggs. But egg recognition also plays a role in some avian obligate brood parasites' reproductive strategy through the pecking of already present eggs in the hosts' clutch to reduce nestmate competition with the parasite's own hatchling. Here we tested egg shape recognition in this parasitic egg-pecking context by exposing two different series of 3D printed models to captive obligate brood-parasitic shiny cowbirds (*Molothrus bonariensis*) in artificial nests. Natural egg-shaped models were pecked more often compared with increasingly thinner models, but there was no effect of increasing angularity on pecking rates, implying that a natural, rather than an artificial, range of variability elicited adaptive responses from parasitic cowbirds.

## 1. Introduction

Eggs are central in avian reproduction, as all birds produce eggs to propagate. Once laid, eggs are typically protected and incubated in nests until they successfully hatch. There are three major exceptions to this pattern: (i) the rejection of brood-parasitic eggs from the nests of many host species [1–3], (ii) the pecking and destruction or the removal of eggs by some brood parasites in hosts' nests [4,5], and (iii) the predation and consumption of eggs ([6]; not the subject of this study).

In the first scenario, viable parasitic eggs are eliminated from the nest of hosts by recognizing and then piercing, grasping, or otherwise removing (e.g. kicking) them [7–9]. Typically, egg rejection is an outcome of host–parasite arms races, whereby the cost of brood-parasitic propagules is ameliorated by host egg rejection, which in turn selects for more mimetic parasitic egg and increasingly fine-tuned host egg-recognition systems. Thus, egg rejection by hosts can be cognitively complex tasks, because even though own eggs are beneficial for fitness and parasitic eggs are detrimental, the shape and coloration of parasitic eggs may be similar to the host eggs through mimicry or deceptive through crypsis [9]. The former is the case of the common cuckoo (*Cuculus canorus*), regarding its different parasitic gentes (host races), which mimic the colour and maculation patterns of the eggs of their respectively host species [10]. By contrast, cuckoo gentes do not mimic host egg shapes [11], and fine-scaled egg-shape recognition is not seen in the rejection of foreign eggs either by parasitic cuckoo or brown-headed cowbird (*Molothrus ater*) hosts [3,12]. Nevertheless, rejecter hosts are more likely to remove grossly non-egg-shaped objects from their nests [13] even when these are mimetic in coloration [14].

In the second scenario, egg-pecking by brood parasites targets non-self eggs to force the host to renest or to reduce competition between the parasite's and host's hatchlings in the same brood [15]. This behaviour is seen when brown-headed cowbirds (*Molothrus ater*) find a nest late in the incubation and destroy most of the host clutch to induce them to start another breeding attempt, thereby generating a new opportunity to parasitize (e.g. nest farming hypothesis; [16,17]). Egg-pecking is also used by parasitic shiny (*M. bonariensis*), screaming (*M. rufoaxillaris*), and bronzed (*M. aeneus*) cowbirds to decrease competition for parental provisions between parasite's and host's nestlings, as in these species the parasitic chicks must compete for food with host chicks (competition reduction hypothesis; [18–22]).

In the case of shiny cowbirds, females show an adaptive egg-pecking behaviour that differs according to the strength of the eggshell [23,24]. Wild-caught females that face clutches with two eggs that differ in eggshell strength, peck more frequently the egg with the weaker shell, thereby increasing the probability of successful puncture [24]. Female shiny cowbirds also show a more intense egg-pecking behaviour when facing larger host clutches, which represent a later stage in host laying and a potentially asynchronously late parasitic hatching [23]. Parasitic eggs laid in such nests might hatch later than host eggs and the foreign nestlings would suffer a further disadvantage in food competition, especially when their host nestmates are older and larger, making clutch reduction all the more relevant for the parasitic chick's survival [25–28]. The shiny cowbird's egg-pecking behaviour is performed mainly by females during the laying visits to host nests, and puncturing is followed by the laying event. There are also puncturing visits by females in which only the puncturing of eggs occurs [27,28]. Nevertheless, there are a few records of males puncturing eggs in host nests in the wild [29–31], and Llambías *et al*. [32] found that males and females in captivity pecked artificial eggs with a similar frequency and intensity. Finally, other, non-parasitic birds are also known to peck eggs and terminate the nesting attempts of con- or hetero-specifics as potential territorial competitors for nest and food resources (e.g. several wren species in the family Troglodytidae; [33–35]).

Here we focus on the shape of objects that are targeted for pecking by captive obligate brood-parasitic shiny cowbirds (hereafter: cowbirds) in the nests of a simulated host. Previous works, using quasi-continuous series of 3D printed models, increasingly deviating from egg-like shapes along one of two axes of variability (width or angularity, see Methods), studied egg rejection by two hosts (barn swallows *Hirundo rustica* [13] and American robins *Turdus migratorius* [14]) of brood parasites (common cuckoos and brown-headed cowbirds, respectively). The authors described in detail the shape-specific thresholds beyond which objects are no longer treated as eggs in the nest (i.e. they are indiscriminately eliminated as potential detritus from the nest cup).

By contrast, here we use this same methodology and series of objects but test, for the first time, for egg-shape recognition in the second specific scenario from above: namely, egg pecking by the brood-parasitic shiny cowbird. Our predictions parallel the findings of previous studies in that we expect more pecks delivered to more egg-shaped objects along both series of object-shape variability [14]. Preliminary trials showed that experimental subjects both pecked and/or grasped models depending on the shape, so we quantified both egg-pecking and egg-grasping behaviours. For the latter, however, we predicted that grasping would be more common when exposed to narrower models which are physically easier to hold but would not depend on the angularity of objects where the models all had the same object width.

## 2. Methods

**Figure 1.**
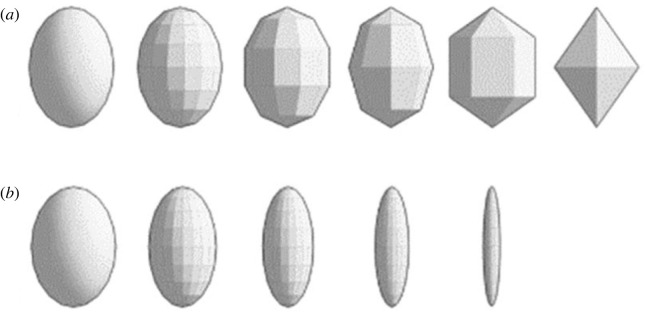
Designs of 3D printed model eggs used in the experiments (after [14]). Models that differ in (*a*) angularity (mm): 0 (control), 4.8, 7.4, 8.8, 11.7 and 12.6, and (*b*) width (mm): 17.1 (control), 13.9, 10.3, 6.9 and 3.1.

We used 3D printed model eggs to assess how cowbirds perceived and behaved in response to gradual changes from more to less egg-shaped traits. We also used a control egg 22.8 mm in length and 17.1 mm in width (similar in size to shiny cowbird eggs; 23.2 ± 0.1 mm in length and 18.7 ± 0.1 mm in width, [36]). We used two different semi-continuous sets of eggs of decreasingly egg-shaped models: (i) five models for the ‘angularity' series that varied in the number and dimensions of flat panels while maintaining the width and length given (i.e. variability in angularity, figure 1*a*), and (ii) four models for the ‘width' series that decreased in width but not in length relative to control egg (i.e. variability in width, figure 1*b*).

For our control model, we used the 3D printed eggs from www.Shapeways.com: catalogue ID no. Cow Bird. The other model eggs were produced using the custom-made services of www.Voxel-Magic.com [14].

The experiments were carried out during the austral spring breeding season, in Buenos Aires, Argentina, in November 2020. We tested twelve 1-year-old shiny cowbirds (seven females and five males). The sample size was based on similar published studies on egg-pecking behaviour of shiny cowbirds [24,32]. These cowbirds were sourced as nestlings from host nests (five from chalk-browed mockingbird *Mimus saturninus* and seven from house wren *Troglodytes aedon* nests) at our study site located at the Reserve El Destino, in Buenos Aires Province, Argentina (35°08′ S, 57°23′ W). They were hand-fed with a protein-rich diet until independence and then kept in a shared outdoor aviary of 63 m^2^ on the campus of the University of Buenos Aires. During the experiments, cowbirds were moved from the aviary to individual cages sized 120 × 40 × 40 cm and located in an indoor room, where they were visually but not acoustically isolated from other subjects.

Each cage was divided into two parts with an opaque partition, and in one part we housed the bird with food and water ad libitum. In the other part, there was an artificial open cup nest, 10 cm in diameter, with dried fibre lining, in which we sequentially put the model eggs. The experimental trial started with the removal of the partition to allow the cowbird to approach the nest and inspect the model egg and was finished when we replaced the partition. Experimental trials were carried out between 09.00 and 11.00 and lasted 17 min on average. Each cowbird had up to three daily trials, with additional trials on consecutive days, until we completed the tests with the 10 different egg models. The order of presentation of the egg models was random, and the subjects experienced each model once; if an individual did not peck or grasp the model at all during a trial, it was repeated up to three times. Trials were recorded with a microcamera connected to a video recorder (Lawmate PVR-1000 or PVR500 ECO).

**Figure 2.**
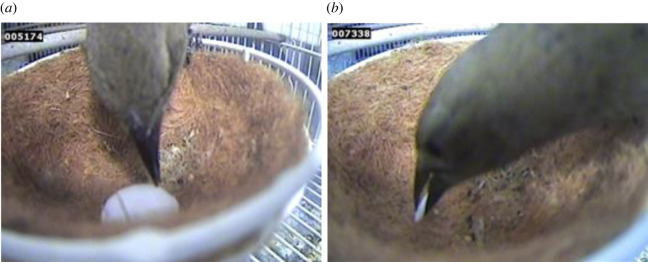
Female shiny cowbirds during the experimental sessions (*a*) pecking a 3D control model egg and, (*b*) grasping the thinnest 3D model egg.

We considered model pecking behaviours as egg-directed responses to the stimuli presented in the study. Therefore, from the recorded videos, we analysed the pecking behaviour of cowbirds by measuring two response variables: (i) the number of pecks (figure 2*a*), and (ii) the number of grasps that each cowbird directed at the model in the artificial nest (figure 2*b*).

### 2.1. Statistical analyses

We analysed the data of each model series (angularity and width) separately adding to each series the data from the control egg. We evaluated how the predictor variable (angularity or width) affected the two types of response variables (the number of pecks or grasps). In each statistical model, we included the duration of the trial (continuous variable, mean ± s.e.: 17 ± 3 min, range: 13–26 min) and the sex of the individual (binomial variable) as covariates. Cowbird identity was included as a random factor, as each cowbird was tested for all the model eggs. For these analyses, we constructed generalized linear mixed models (GLMM). We used a within-subject design, which is a powerful approach, and the level of replication (*N* = 12 subjects) is appropriate for modelling individuals as a random effect [37].

For the angularity series, we used models with negative binomial distribution and log link function when analysing the number of pecks and number of grasps. For the width series, we used: (i) the hurdle model with Poisson distribution and logit link function when analysing the number of pecks, and (ii) the hurdle model with negative binomial distribution (due to overdispersion for the Poisson model) and log link function when analysing the number of grasps. The hurdle model is used for count data that is zero-inflated [38] and has two parts: the first describes the probability of a zero count and the second describes the expected rate of the non-zero counts.

Statistical analyses were carried out using R software, v. 4.0.0 [39] and the R Studio, v. 1.0.143 [40]. We performed GLMM analyses using the glmmTMB R package [41]. All tests were two-tailed, values are reported as means ± s.e., and differences were considered significant at α ≤ 0.05.

## 3. Results

### 3.1. Width series

**Figure 3.**
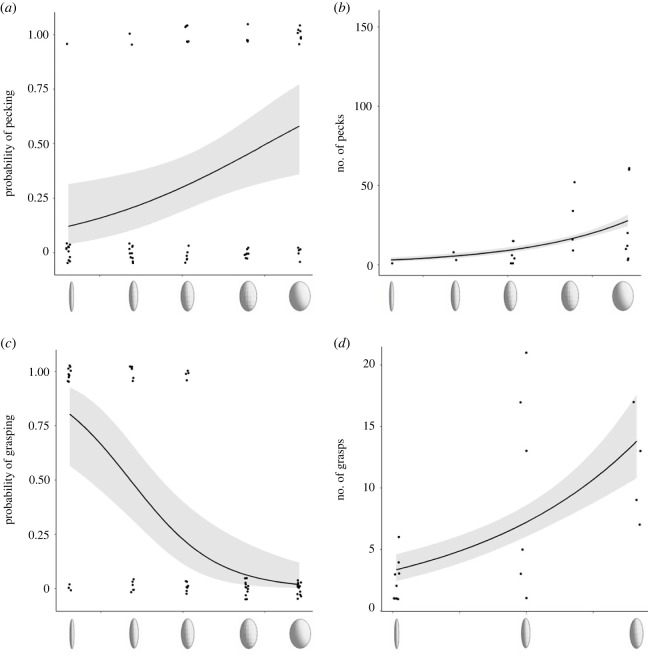
Results of the width series: the probability of pecking and the number of non-zero pecks increased with the width of the eggs; (*a*) plot of a logistic regression of the variable pecks on width, where pecks is a variable that is equal to 1 if the number of pecks is greater than 0, and 0 if number of pecks = 0; (*b*) plot of the Poisson regression considering only the data with the number of positive pecks. The number of grasps (*c*,*d*): narrower objects had a higher probability to be grasped, but the number of non-zero grasps increased with the width of the objects; (*c*) plot of a logistic regression of the variable grasps on width, where grasps is a variable that is equal to 1 if the number of grasps is greater than 0, and 0 if number of grasps = 0; (*d*) plot of the Poisson regression considering only the data with the number of positive grasps. Lines represent regressions and the shaded areas indicate the standard errors. Points have been jittered on the x-axis in all figures and also on the y-axis in (*a*) and (*c*), to avoid overlap.

Results of the width series showed that both the probability that a peck occurred and the number of non-zero pecks both increased with the width of the eggs (table 1, figure 3*a,b*). The probability of pecking was not affected by the length of the trial. Sex predicted the probability of pecking, with males showing a lower probability than females (0.32 ± 0.07 versus 0.61 ± 0.06), whereas there were no differences between the sexes in the number of pecks (table 1).

**Table 1.** Results of the hurdle model for the probability and number of pecks. The among-individual variance is mean: 0.81, s.d.: 0.90.

	estimate	s.e.	*Z*-value	*p*
zero-inflation model				
intercept	0.457	2.616	0.175	0.861
width	−0.180	0.068	−2.644	<0.01
duration	0.001	0.002	0.692	0.489
sex (male)	1.354	0.660	2.050	0.040
conditional model				
intercept	−1.704	0.856	−1.991	0.046
width	0.220	0.020	11.015	<0.0001
sex (male)	−0.355	0.681	−0.522	0.602
duration	0.001	0.001	1.781	0.075

The probability that grasping occurred and the number of grasps that cowbirds performed also differed across the width series models (table 2). Narrower objects had a higher probability to be grasped but the number of non-zero grasps increased with the width of the objects (figure 3*c*,*d*; table 2). Length of the trial and cowbird sex did not affect the probability and number of grasps (table 2).

**Table 2.** Results of the hurdle model for the probability and number of grasps. The among-individual variance is mean: 9.33 × 10^−10^, s.d.: 3.06 × 10^−5^.

	estimate	s.e.	*Z*-value	*p*
zero inflation model				
intercept	1.426	2.901	0.491	0.623
width	0.479	0.136	3.517	0.0004
sex (male)	1.665	0.897	1.856	0.063
duration	−0.005	0.003	−1.676	0.094
conditional model				
intercept	2.687	3.030	0.887	0.375
width	0.245	0.070	3.512	<0.001
sex (male)	−0.331	0.414	−0.800	0.423
duration	−0.002	0.003	−0.847	0.397

### 3.2. Angularity series

**Figure 4.**
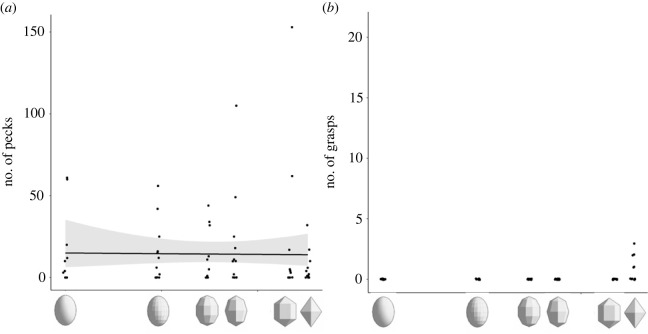
Results of the angularity series: the probability of pecks did not vary with the angularity of the model (data not shown); (*a*) plot of the number of pecks: the angularity of the model did not affect the number of pecks; (*b*) plot of the number of grasps: cowbirds did not grasp these objects, except for the model category of the larger panel length (no statistical test). Line represents regression and the shaded region indicates standard errors. Points have been jittered on the x-axis to avoid overlap.

Results of the angularity model series showed that this variable did not affect the number of pecks (table 3, figure 4*a*). Similarly, the length of the trial or the sex did not affect the number of pecks (table 3). Regarding the number of grasps, cowbirds did not grasp these models, except for the category of the larger panel length in which only five cowbirds grasped the model egg (figure 4*b*, table 3). Therefore, we could not carry out a statistical analysis. The duration of the trial and cowbird sex did not have a significant effect on the number of grasps (table 3).

**Table 3.** Results of the negative binomial model for the number of pecks. The among-individual variance is mean: 3.39, s.d.: 1.84.

	estimate	s.e.	*Z*-value	*p*
intercept	2.08	1.95	1.07	0.29
panel length	−0.07	0.04	−1.43	0.15
sex (male)	−1.39	1.17	−1.18	0.24
duration	0.0006	0.002	0.39	0.70

## 4. Discussion

We used two quasi-continuous series of models with variation along a width or an angularity axis from egg to non-egg shape [13] to analyse the limits of egg recognition in the context of egg-pecking by captive brood-parasitic cowbirds in simulated host nests. We found that decreasingly egg-shaped objects along the width axis received fewer pecks and more grasps, whereas there was no such relationship between increasingly angular objects and the pecks or grasps they received. This was in part unexpected because both width and angular axes generate increasingly non-egg-shaped objects with the width series generating thinner objects that retained the length but not the width of the original egg-shaped object, whereas the angularity series generating more angular objects that retained both the length and the width of the original egg model. Finally, as seen in nature, captive female cowbirds showed a greater probability to peck eggs than captive males when shifting along the width dimension.

These results are consistent with the hypothesis that variation in natural traits (egg width) is more salient for egg recognition than variation in artificial traits (egg angularity). The result that thinner models were more frequently grasped than wider ones suggests that the decreasing width dimension may also indicate a potential food source, (i.e. insect larvae) rather than an egg phenotype. Accordingly, models in the angularity series were also consistently and intensively pecked (independent of dimensions) and rarely ever grasped, implying that if width and length retained their egg-like dimensions, the shape of the object does not matter for egg recognition by parasitic cowbirds, despite the unnatural edges of the increasingly angular models. Along this angularity dimension, we found that parasitic cowbirds were less discriminatory than egg-rejecting swallow and robin hosts [13,14], which could be due to foreign egg rejection being a more fitness-critical trait for hosts of brood parasites than host egg-pecking for parasites themselves. Given that we are contrasting a series of egg-like models with relatively small changes in shape, we interpret similar levels of pecking as similar degrees of egg recognition. Nevertheless, this is an assumption that we cannot test directly and for the furthest non-egg-shaped objects we cannot make the claim that they were still pecked because of their egg-like qualities. Moreover, the analysis of egg grasping behaviour versus angularity would have benefited from a larger sample size due to low signal to variance ratio (see Results).

By rejecting the wrong egg, hosts risk engaging in double jeopardy: (i) reducing their fitness by eliminating one of their eggs, and (ii) still paying the costs of incubating and raising a foreign egg and chick in the nest. By contrast, parasitic pecking non-eggs accidentally in the nest does not seem to carry a similarly severe fitness penalty, except for the time wasted on it and potentially being exposed to more harmful physical attacks by the nest owner attacking the parasite [42]. For example, during the laying visits of shiny cowbirds to chalk-browed mockingbird nests, the parasitic female pecks quickly and repeatedly the already present eggs, whereas the hosts deliver blows with their beak to the parasite's head and body [23,42]. Cowbird nest visits in nature are very short (20 s on average), they dedicate a few seconds to frantically pecking the eggs, and in most cases, they manage to lay their eggs and fly away without injuries [42]. Therefore, cowbird females could be best suited to peck any object whose width and length are similar to that of an egg, whereas other variables, such as angularity, do not represent the natural variation in egg characteristics and do not attract decreased responses by the parasite. To test these predictions, we performed *post hoc* tests evaluating whether latency to first egg pecking varied with angularity or width. We found that although the width was positively related with the latency to peck (GLMM, negative binomial test, intercept: estimate 3.09, s.e.: 2.37, *Z*-value: 1.31, *p*: 0.19; width: estimate: 0.16, s.e.: 0.06, *Z*: 2.77, *p*: 0.006; among-individual variance, mean: 1.71×10^−0.8^, s.d.: 0.0001), angularity did not affect the latency to peck (GLMM, negative binomial test, intercept: estimate 5.90, s.e.: 1.00, *Z*-value: 5.89, *p* < 0.0001; angularity: estimate: −0.05, s.e.: 0.03, *Z*: −1.57, *p*: 0.12; among-individual variance, mean: 0.20, s.d.: 0.45). The long trial durations of the study may affect pecking decisions relative to natural conditions. Nevertheless, our video studies, both published and unpublished, indicate that the relatively short period of pecking at natural nests is not because the cowbirds themselves are not interested in pecking the host eggs longer but because they are typically attacked and physically displaced by the larger (mockingbird) hosts from their respective nests. We, therefore, consider the longer duration of our trials biologically relevant as the cowbirds were motivated to respond throughout.

It could be thought that as cowbirds egg-pecking behaviour occurs before they lay the parasitic egg into the host nest, they can gain benefits by pecking any egg. In these cases, the egg recognition might be not favoured by selection. Nevertheless, if time is of the essence, because of potential harm from hosts, we would predict that egg recognition would be nearly instantaneous instead of indiscriminate, as pecking non-egg-shaped objects (detritus, etc) in the nest would be disfavoured by selection. Moreover, a previous study in which shiny cowbirds were filmed visiting chalk-browed mockingbird nests, showed that the egg-pecking behaviour varied depending on the strength of the eggshells in the clutch, supporting the idea that egg recognition is an adaptive behaviour favoured by selection [23].

Overall, using the same methodological advances and treatments as applied before [13,14], we found that brood-parasitic shiny cowbirds are less discriminatory in egg recognition compared with hosts of brood parasites, as they modulated their behaviours along the width but not along the angularity dimension of egg-shaped object models. Whether experiments in the field parallel these behavioural dichotomies seen in captivity and what fitness costs misdirected pecking may entail, remain to be analysed using active nests of hosts, shape-series model egg-like objects, and video-recordings of the responses of wild cowbirds at natural host nests.

## Ethics

All procedures involving in this study were permitted by Organismo Provincial para el Desarrollo Sostenible, Argentina (permit no. 71/16- O.P.D.S.), comply with the current laws of Argentina and have been approved by a local ethics review committee (permit no. 148 b - CICUAL).

## Data Availability

Research data supporting this publication are available from the Figshare repository located at: Experimental Data: 10.6084/m9.figshare.22400983 [43] and 10.6084/m9.figshare.22400809 [44]. R Code: 10.6084/m9.figshare.22400512 [45].
